# A Review on Effectiveness of Plasma Therapy in Severe COVID-19 Patients

**DOI:** 10.7759/cureus.28914

**Published:** 2022-09-07

**Authors:** Kartik Kapil, Pramita Muntode Gharde

**Affiliations:** 1 Department of Community Medicine, Jawaharlal Nehru Medical College, Datta Meghe Institute of Medical Sciences (DU), Wardha, IND; 2 Department of Community Medicine, Jawaharlal Nehru Medical College, Acharya Vinoba Bhave Rural Hospital, Datta Meghe Institute of Medical Sciences (DU), Wardha, IND

**Keywords:** corona virus, sars-cov2, convalescent plasma therapy, rt pcr, covid 19

## Abstract

Coronavirus 2019 has created a big threat to the modern world. Many researchers and scientists had taken the burden of finding information about this entity, its structure, its transmission, and also about the treatment that can be given to individuals infected by it. There has been use of different medicines at different times simultaneously researching about them, starting with only symptomatic and supportive treatment, then antimalarial agents like chloroquine and hydroxychloroquine, then going to favipavir, and other antivirals, then came the use of vaccines and also convalescent plasma therapy for COVID-19. The most advanced is convalescent plasma use for the treating coronavirus. Using plasma of patients who have remitted from this disease and putting it into those individuals who are dealing with the disease or are critically ill for improvement of their health status. This treatment has been used for many other diseases too and has been proven efficacious. So, this technique is being used and studied for coronavirus 2019 as well. There have been set certain criteria for those who can donate plasma and also criteria for the recipients of this technique. Also, there can be adverse reactions or even side effects with this, like transfusion-related acute lung injury (TRALI), so they should also be kept in mind during treatment with this method. So, though there are many methods to date to treat these individuals but one of the latest ones is using plasma, which is proven to be efficacious but still many studies are under process for the same.

## Introduction and background

By the end of 2019 December, a new viral strain started spreading havoc all over the globe. This started in a city in China called Wuhan. This is basically a viral element that is made of RNA, and due to its similarity with the SARS virus, it was known as SARS-CoV-2 or basically coronavirus 2019. Individuals affected by this entity presented with a number of symptoms including cough, cold, dyspnoea or even symptoms of pneumonia to life-threatening conditions [1]. Since this was creating such a fearful environment for everyone, the government took up some measures to limit this transmission which was spreading via aerosols. People were asked to stay indoors, the lockdown was imposed in various countries, it was essential to cover your face, and also proper hygiene practices came up to limit this disease [2].

Apart from these, research we are done to find an apt medicine to treat people who had already gotten infected by the virus. Treating a patient started with normal supportive management of the patient and also symptomatic treatment [3]. Then came the prevalence of the use of many antivirals, steroids, malaria drugs, etc. [4]. One of the latest research projects was the advent of convalescent plasma for treating these individuals. This basically means using of plasma of individuals who already remitted from corona and giving that plasma to someone infected now or in a serious condition [2].

## Review

COVID-19

In December of 2019, in Wuhan in China, a novel coronavirus called as SARS-CoV-2 started spreading infection, which was soon called to be a pandemic by WHO, i.e., World Health Organization. This coronavirus originated from a bat virus and is basically an RNA virus that has recently become a large-scale public health problem affecting a number of individuals all over the world. The broad family of single-stranded RNA viruses called as the Coronaviridae includes both human and animal viruses. Phylogenetically closely linked (88%) to two bat-derived SARS-like coronaviruses discovered in 2018 in China: less similarity with MERS-CoV (50%) and SARS-CoV (79%). It is a part of the Sarbecovirus subgenus, beta-coronavirus [1]. According to the data by end of October 2020, there were around 44 million COVID-19 cases throughout the world with more than 1.18 million mortalities [2]. The four structural proteins of COVID-19 are nucleocapsid (N), Envelope (E), membrane (M), and spike (S). The S protein, which is a receptor for COVID-19 and SARS-CoV-2, is highly similar to the ACE2 receptor in human cells [3]. Myocardial pericytes, capillary artery and venous endothelial cells, smooth muscles of arteries, respiratory epithelial cells, [4] and the intramyocardial microvascular network all contain a significant amount of ACE2 receptor [5-7]. Due to these specific cell localizations, endothelial function deficits consistent with COVID-19 organ system preference substantially intermediate clinical course. Clinical observations showing a typically deteriorated course in patients with underlying comorbidities, like metabolic, respiratory, malignancies, and cardiovascular, provide additional evidence for this [8].

COVID-19: Signs and Symptoms

Coronavirus spreads via droplet infection from person to person, which can transmit via talking, coughing, sneezing, or even singing. It is proven that it can spread through the environment via aerosols [9]. The period of incubation for this particular disease is found to vary between two days to around two weeks [10]. If we see at the symptoms, then the individual usually presents with very general common cold or flu type symptoms like fever, sore throat, cough, sneeze, dyspnoea, etc. and because of which it was earlier mistaken to be flu but later on identified as coronavirus. Other clinical manifestations can take the form of anosmia, a sore throat, fever, muscle weakness, exhaustion, headache, or diarrhoea [11]. The evidence of developing viral pneumonia is usually found after two weeks of the disease, with evident radiological findings especially the ground glass opacities, alveolar exudates, patchy consolidation, etc. [12]. In the case of cytokine storm, unbalanced and disproportionally uncontrolled immune reaction rises to the level of burst as a result of external factor like COVID-19, particularly disadvantaged clinical course with increased death rate occurs [12]. So, symptoms of this disease vary from mild flu-type disease to severe lung involvement that can lead to mortality [2]. The mortality rate with this virus is around 2%. And because of this increased rate, there is a very urgent need to look into controlling this situation before it turns morbid [9].

The government is trying its best to limit the transmission rates, and even has got positive results for the same. Some of the interventions include keeping a social distance among people, always covering your nose and mouth with a mask or a cloth, and also keeping good personal hand hygiene practices [9]. These are the preventive measures taken by the government, but those already infected need to be vigorously treated as well. For that, different research is going on all around the world and many methods have been formulated for treatment. The basic plan is to give supportive and symptomatic treatment to the patient including fluid management and proper oxygenation. But the scientists are reluctant to find a drug that is most potent against this virus [9].

COVID-19: Treatment

SARS-CoV-2, which is causing COVID-19, has become widespread worldwide, and there is currently no cure [13]. Physical separation, isolation, and other sterilising practises have shown some effectiveness in decreasing the pandemic, but most nations are far from being free of it. Treatment involves the use of antiviral drugs such chloroquine, ribavirin, and remdesivir, hydroxychloroquine [14-18]. Azithromycin is one of many antibiotics that tackle various illnesses. It has been investigated how hydroxychloroquine and Azithromycin work together [15]. The following therapy by combining azithromycin and hydroxychloroquine on six patients, Gaurtred et al. [16] reported that the patients' nasopharyngeal swabs were 100% virus-free. The host's inflammatory reactions are suppressed by corticosteroids, and they stop an overactive inflammatory response. Corticosteroids may, however, have negative side effects, such as prolonged hospital stays without a reduction in mortality, when used in conjunction with viral therapy [17]. Chloroquine, which is an antimalarial medication, and azithromycin, an antibiotic, have shown promise against COVID-19; however, their efficacy is since been questioned [18]. Ritonavir, remdesivir, favipavir, ribavirin, tocilizumab and sarilumab, are some of the other prospective treatment drugs that have been explored thus far, but their effectiveness requires additional research [15-19]. Given the condition of poor and emerging nations, where many individuals cannot afford ventilators or to lock themselves up for long periods of time, the epidemic should be halted so that healthcare can cope. Regionalized herd immunity can help reduce the spread of disease in susceptible areas in the absence of a recognised therapy and tested vaccination [20].

In particular, two methods of repurposing common medications and developing innovative therapeutic pharmaceuticals are being investigated: preventing viral entrance into host cells and suppressing different processes in viral reproduction in the cells. Coronaviruses attack ACE-2 receptors which are highly expressed inside the cells of the human body's lung and gastrointestinal tissues. The receptor-binding domain (RBD) of coronavirus spike (S) protein interacts with ACE-2 receptors over the plasma membrane of cells that are infected, causing receptor-mediated endocytosis. Three techniques are now being tested in an attempt to prevent infection by blocking this connection [21]. Favipiravir inhibits the activity of RNA replicase and viral RNA polymerase. Remdesivir stops viral replication by interfering with the activity of RNA polymerase of the virus and reducing the synthesis of viral RNA. Lopinavir inhibits the viral proteases action. Ritonavir inhibits the action of viral proteases. Chloroquine prevents ACE-2 from being glycosylated. Ribavirin inhibits the activity of viral RNA polymerase. Umifenovir (Arbidol) prevents viral entrance into host cells by inhibiting membrane fusion [21].

In extreme circumstances, plasma therapy might be the most important tool in the war against COVID-19. Surviving individuals play an important role in both herd immunity and plasma transmission in this situation. All surviving individuals should be well-documented in order to exploit their potential. Their convalescent plasma must be tested for neutralising antibodies and preserved in blood banks around the world, ready to be given when needed [20].

Convalescent plasma therapy

Convalescent plasma, or the plasma from those people who have already recovered from COVID-19 illness, is an already used therapy in other infections. In a pandemic, convalescent plasma may be a readily available supply of antiviral antibodies [9]. In COVID-19 illness, it may have a variety of positive effects. The evident explanation, first is that antibodies present in convalescent plasma can reduce viral load in blood. The delivery of convalescent plasma at an initial phase of the illness, equivalent to the procedures used during the SARS pandemic, would potentially be more successful [22].

In the majority of viral diseases, viral load peaks in the first week after illness, and a main immune response of the host is generally formed by the 10th to 14th day of infection [23,24], signifying the virus's clearance. Antibody-dependent cellular cytotoxicity, complement activation, and phagocytosis (ADCP) are further possible pathways [25]. Second, the existence of non-neutralizing antibodies that bind to pathogenic material might be beneficial [26]. The principle of passive immunization underpins the use of convalescent plasma, in which individuals get plasma that is rich in antibodies from people who have remitted from the disease. Convalescent plasma, in contrast to active immunisation, which is often used to avoid infection, could be used to avoid disease. The major goal of convalescent plasma, although is to heal people with severe illnesses. Convalescent plasma is thought to work as a treatment option in a variety of ways. IgG and IgM antibodies in convalescent plasma may attach to the particular pathogen (SARS-CoV-2, MERS-CoV, SARS) and serve as neutralising antibodies, inhibiting the virus [27].

There are several benefits and drawbacks of transfusing convalescent plasma. Convalescent plasma transfusion has been linked to a short-term immunity and can be used as a quick and effective treatment. Convalescent plasma, if administered early enough, should be effective in cases of moderate or severe infection. Transfusion of infectious organisms, transfusion-associated circulatory overload (TACO), transfusion-related acute lung injury (TRALI), and antibody-dependent enhancement are all potential dangers of convalescent plasma transfusion (ADE). Due to the existence of non-neutralizing antibodies and even neutralising SARS-CoV antibodies, ADE is a possible worry that might lead to severe tissue damage which is immune mediated [27].

Convalescent plasma treatment is successful in reducing death rates and has a considerable influence on modifying the immune system and reducing the load of the virus. Based on the duration of stay in the hospital, convalescent plasma treatment appears to have the potential to abbreviate the course of illness and aid patient recovery. Convalescent plasma infusion has a low chances of significant side effects, which are largely manageable [28]. Convalescent plasma treatment may benefit immunodeficient patients. However, it is important to remember the risk [29]. It is possible for plasma to be contaminated, and this could be problematic for someone with immunodeficiency. According to Hähnel et al., every blood product used to treat COVID-19 should go through stringent testing to verify its high quality and safety [30].

SARS-CoV-2 and SARS-CoV have recently been discovered to attach to the same entrance receptor (ACE-2) with equivalent affinity, in addition to being highly pathogenic coronaviruses with lung tropism. SARS-CoV polyclonal Ab also prevents SARS-CoV-2 spike glycoprotein (S)-mediated cell entrance [24]. To guarantee effective antibody titres and a surge in the patient's immune response in a timely fashion, collection, and treatment with CP should be done at the appropriate time. Early CP therapy has been demonstrated to have better clinical results than delayed interventions in trials. Before antigen triggers the major immune response, there is a 10-day incubation phase. Low-affinity IgM antibodies are generated later, followed by low-affinity IgG antibodies, which peak on day 21. Only as a subsequent reaction may high-affinity IgG antibodies be generated fast (in 3-5 days). As a result, CP must be administered early in the disease course, when the body has not yet generated IgG antibodies. Passively infusing high-level, high-affinity IgG can strengthen the humoral immune response, minimise the immune system's recurrent activation of killer T cells, avoid cytokine storms, and keep the illness from developing to a critical stage at this time [28].

Convalescent Plasma Therapy: Procedure

Convalescent plasma donors - between 18 and 60 years old, clinically and laboratory-confirmed recovered COVID-19 patients were chosen as convalescent plasma donors. Female donors having a history of pregnancy were excluded from the study to prevent TRALI. At the time of donation, selected donors had negative RT-PCR for COVID-19 and other conventional virology tests, despite the fact that their COVID-19 test results had previously been positive by RT-PCR. Also, minimum of 14 days prior to donation, all donors should be free of COVID-19 infection signs. They were requested to fill out associated plasma donation and permission paperwork after being questioned and assessed by a trained physician. The total volume of plasma collected is 500mL. Donated plasmas had an antibody titre cut-off index of more than 1.1, according to laboratory tests and it is kept in the freezer until the result of the screening tests were released. After it has met the eligibility criteria for transfusing, the donated plasma is then sent to blood banks.

According to the FDA, those patients who met the following criteria, can use CP as a treatment- (i) Age ≥ 18 years; (ii) Confirmed COVID positive status from the laboratory; (iii) Severe or immediately life-threatening COVID-19.

The severity of disease is measured by (i) dyspnoea; (ii) tachypnoea ≥ 30/min; (iii) saturation of oxygen in blood ≤ 93%; (iv) PaO_2_/FiO_2_ < 300; (v) Infiltrates in lungs >50% within 24-48 hours; and the disease is considered life-threatening if there is a respiratory failure or septic shock history.

Criteria for excluding some individuals from this trial include (1) patients who are either on ventilatory support or are intubated; (2) those undergoing kidney or any liver disease; (3) individuals under septic shock; (4) even if the physician treating the patient thinks it is not good for the patient; (5) someone who has plasma hypersensitivity [31].

Convalescent Plasma Therapy: Treatment for COVID-19

Convalescent plasma is also proposed for the treatment of severe coronavirus pneumonia. From start, the COVID-19 pandemic had a large fatality rate because of a lack of adequate treatment for very sick people. A total 396 plasma donations were collected from 277 convalescent donors. Per 10 kg weight of the body, patients were given plasma with IgG content of 0.7-0.8. The primary goal was to survive the first 28 days; 77% were male, aged 54 and 15.6 years (range 27-85), with a body mass index of 29.7 4.4; hypertension was reported in 39% of the participants, and diabetes was reported in 20.7%; 19.5% had an immunosuppressive condition, and 23% were health workers. Plasma was given to 54 patients (63%) who were spontaneously breathing via oxygen support and 31 patients (37%) who were on mechanical ventilation. The 28-day survival rate was 80%, and 90% of patients were breathing spontaneously, and 62% were on mechanical ventilation. The pneumonia clinical scale by WHO improved significantly at seven and 14 days, as did PaO_2_/FiO_2_, ferritin, and LDH in the week after the infusion. A modest case of circulatory volume overload and a feverish response were detected. Plasma infusions for convalescents are practical, safe, and possibly helpful, especially before mechanical breathing is required. They are a promising therapeutic alternative for treating COVID-19 severe cases until more effective treatments become available [32].

According to research most of patients improved their symptoms after getting convalescent plasma transfusion, involving body temperature normalization, different degree of lung lesion absorption, ARDS resolution, and weaning from ventilator between one day to 35 days. After patients got CPT at various dosages, all trials found that there was no mortality. However, it remained unclear if the large number of patients who survived was due to the use of numerous additional drugs or CPT therapy, or a combinational/synergistic impact of both.

Our findings were hampered by a scarcity of high-quality RCT trials and related literature. Most of the studies that were described were mostly case reports, lacked suitable control groups, and had a moderate to high risk of bias [33]. Transfusion-transmitted infection is the initial source of worry [34]. The second point of worry is TRALI, that can be fatal in individuals who have previously been diagnosed with ALI. To reduce the possibility of transfusing anti-HNA/HLA/HPA antibodies from pregnant females, male donors are typically recommended. This might be harmful in COVID-19, where women are proven to be having greater levels of IgG, and anti-HPA/HLA/HLA antibody screenings can be used. Antibody-dependent enhancement (ADE) is a conceptual concern [34]. Specific antibodies that are found in CP can help individuals with deficient humoral immune deficits, and the clotting factors in it can support patients with bleeding diathesis [35].

CP is now being studied as a primary therapy for corona as well as a prophylactic. SARS-CoV-2 and other viruses have showed promise when treated with CP. When developing therapeutic strategies, it's important to consider the time frame of administration in relation to the onset of disease, the time for donation in relation to the resolution of symptoms, the magnitude of the donor's disease, the recipient's pre-transfusion serology, as well as the donor's antibody titres. Measures of success should be customized to the demographic under study [36].

## Conclusions

The greatest worldwide health disaster, the COVID-19 outbreak, requires immediate attention. There are currently no proven therapy alternatives for COVID-19-infected patients who are seriously unwell. In this period when the medicines which are existing are being tested and also vaccines that are being made are under evaluation, then at this time for the treatment of a patient, using plasma therapy is a good alternative. For this therapy to work certain criteria are essential to be followed - firstly, it is important to know who all come under donors who are eligible to donate their plasma. Then there should be the availability of facilities to process and test the blood. Also, to know who proper recipients are to receive this therapy. And lastly what should be the dose given to these recipients?

Given the dearth of knowledge about the COVID-19 virus's natural history, PRT should be considered as an additional layer of protection for convalescent plasma patients. According to research, plasma therapy can be a good therapeutic alternative in addition to antiviral/antimicrobial medicines, with promising indications of being safe, improving symptoms, and decreasing mortality. We understand that a firm conclusion on the appropriate dose and time of therapy points to COVID-19 cannot be reached at this time; hence, massive clinical trials are required to combat the pandemic.
